# Patients with floaters: Answers from virtual assistants and large language models

**DOI:** 10.1177/20552076241229933

**Published:** 2024-02-14

**Authors:** Gloria Wu, Weichen Zhao, Adrial Wong, David A Lee

**Affiliations:** 1 Department of Ophthalmology, 12224 University of California San Francisco School of Medicine, San Francisco, California, USA; 2 University of California, Davis, Davis, California, USA; 3 University of Texas Health Science Center at Houston, McGovern Medical School, Houston, Texas, USA

**Keywords:** ChatGPT, Bard, Google Assistant, Alexa, LLM, virtual assistants, floaters, health literacy, American Academy of Ophthalmology

## Abstract

**Objectives:**

“Floaters,” a common complaint among patients of all ages, was used as a query term because it affects 30% of all people searching for eye care. The American Academy of Ophthalmology website's “floaters” section was used as a source for questions and answers (www.aao.org). Floaters is a visual obstruction that moves with the movement of the eye. They can be associated with retinal detachment, which can lead to vision loss. With the advent of large language model (LLM) chatbots ChatGPT, Bard versus virtual assistants (VA), Google Assistant, and Alexa, we analyzed their responses to “floaters.”

**Methods:**

Using AAO.org, “Public & Patients,” and its related subsection, “EyeHealth A-Z”: Floaters and Flashes link, we asked four questions: (1) What are floaters? (2) What are flashes? (3) Flashes and Migraines? (4) Floaters and Flashes Treatment? to ChatGPT, Bard, Google Assistant, and Alexa. The American Academy of Ophthalmology (AAO) keywords were identified if they were highlighted. The “Flesch-Kincaid Grade Level” formula approved by the U.S. Department of Education, was used to evaluate the reading comprehension level for the responses.

**Results:**

Of the chatbots and virtual assistants, Google Assistant is the only one that uses the term “ophthalmologist.” There is no mention of the urgency or emergency nature of floaters. AAO.org shows a lower reading level vs the LLMs and VA (*p* = .11). The reading comprehension levels of ChatGPT, Bard, Google Assistant, and Alexa are higher (12.3, 9.7, 13.1, 8.1 grade) vs the AAO.org (7.3 grade). There is a higher word count for LLMs vs VA (*p* < .0286).

**Conclusion:**

Currently, ChatGPT, Bard, Google Assistant, and Alexa are similar. Factual information is present but all miss the urgency of the diagnosis of a retinal detachment. Translational relevance: Both the LLM and virtual assistants are free and our patients will use them to obtain “floaters” information. There may be errors of omission with ChatGPT and a lack of urgency to seek a physician's care.

## Introduction

**Figure 1.**
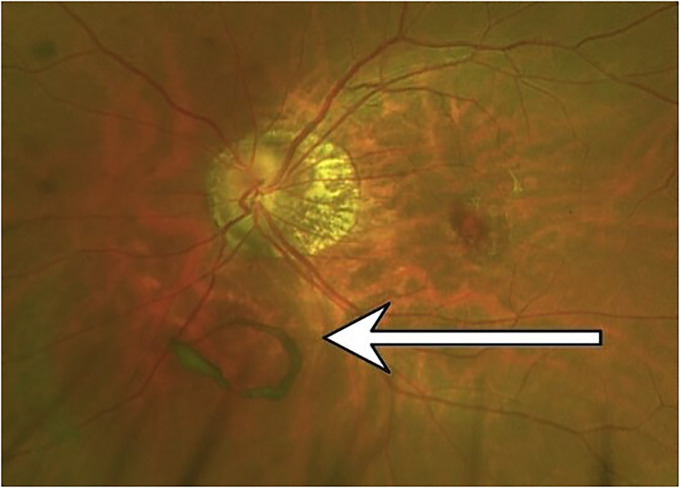
Floater (see arrow).

Floaters are a visual obstruction that moves with the movement of the eye and is prominently seen when looking at a blank page.^1,2^ With at-home work schedules and frequent computer use, floaters can be seen often and are considered frightening by many patients (Figure 1).

As ophthalmologists, we are interested in the query term “floaters” for LLM such as ChatGPT and Bard. Google Assistant and Alexa are virtual assistants and are used for health education as well.^3–5^

Floaters are a common complaint among people of all ages when seeking care with an ophthalmologist or optometrist. The patient demographic for this complaint includes technology workers, students, and computer workers, aged 20–40 years old who are part of the Internet demographic. Older patients also search for “floaters” as well. It is the most common complaint in a retinal practice.

Floaters affect seven out of ten people of all ages.^6–10^ It is a common complaint for new patients when they visit a retinal specialist. In a survey of 603 participants, 76% of respondents saw floaters.^6,9^ The spontaneous and sudden appearance of floaters carries the risk of future retinal holes, retinal detachment, and blindness in certain predisposed individuals with previous retinal disease or high myopia.^1,2,8–10^

Treatment for floaters ranges from monitoring the patient to seeking a retinal specialist for an examination to determine the cause of floaters (retinal hole, vitreous hemorrhage, retinal detachment, transient ischemic attack, or migraine headaches). For patients with no previous eye exam history, the appearance of floaters may be ignored until a significant visual loss occurs, often associated with a retinal detachment.

ChatGPT-3.5 is a free, large language model (LLM) chatbot (OpenAI, San Francisco); it was released on November 30, 2022. Six days later, it had over 1 million users. By January 2023, it had 100 million users. ChatGPT's natural language processing capabilities use artificial intelligence (AI) to perform autocompletion of sentences, thereby creating answers to various queries.^3–5^ Given the word “floaters,” these LLMs may predict the next word to be “retinal detachment” or “flashes of light” based on statistical parameters learned from prior training data. Billions of phrases and the juxtaposition of words are programed into the generation of natural language. Thus, the fluency of the answers results from the mass compilation of sentences and phrases by ChatGPT and Bard's algorithms.

ChatGPT requires high health literacy from its users through the use of high school and college vocabulary sentence structure.^3–5^ Bard uses slightly lower reading-grade vocabulary. One hundred thirty million Americans read at 6th-grade level or below.^7^ Some studies report that the average American reads between 7th and 8th grade.^11–14^ Health literacy is a recognized problem for most Americans as this was highlighted during the COVID-19 pandemic.^11–14^

Bard, the LLM created by Google, was launched on March 23, 2023, but was withdrawn in 24 h from the U.S. market after initial “incorrect” answers.^15^ Bard was relaunched on August 28, 2023, in the United States. ChatGPT has maintained its growth since November 2022.^16^

Google Assistant, is an AI-mediated virtual assistant which can receive verbal questions and/or typed questions in its mobile app. It has 99% of the market share of virtual assistants.^17–19^ The virtual assistant allows for hands to be free and no need for typing on a keypad. Google Assistant was launched in 2016. It has 500 million users with more than one billion voice searches occurring each month in 2022.^17,18^ More than 40% of voice search results are from featured texts from websites. More than 20% of searches on the Google App are done with voice queries.^17,18^ Does ChatGPT or Google Assistant adhere to American Academy of Ophthalmology guidelines for “floaters”?^20^

Alexa is an Amazon-sponsored virtual assistant. It has been less popular than Google Assistant.^17–19^

The American Academy of Ophthalmology (AAO) serves as the official public face of the largest organization for practicing ophthalmologists in the United States.^20^ Its website, AAO.org, provides educational information to the public and ophthalmologists about various eye diseases, e.g., floaters.^20^ The AAO website aims to provide accurate facts, advice, and data, considered important by board-certified ophthalmologists in America. Our study investigated ChatGPT-3.5, Bard, Google Assistant, Alexa, and AAO.org's “Common Eye Diseases and Eye Health Topics,” under the subheading, “Floaters.”

## Methods

From the AAO.org, “Common Eye Diseases and Eye Health Topics,” subheading of “Floaters,”^17^ we used AAO's four questions to query the LLMs and virtual assistants and recorded the responses. According to the AAO website answers, we chose the keywords highlighted with bullet points, bold or in quotation marks, and underlined text. In question 2, there is only one underlined word, “star.” In addition, our two ophthalmologist authors (GW and DAL) selected “flashing lights,” “Lightning streaks,” and “vitreous rubs/pulls on the retina” to be the keywords. There was one video on floaters that was repeated twice on AAO.org and had a closed caption option. Thus the video's transcript was only included once. These captions were transcribed onto Google Docs and were included in the keywords. We chose to use the highlighted keywords to show that these words were considered important to the AAO. The “ophthalmologist” mention was considered important because floaters can lead to retinal holes and retinal detachment, a potential cause for severe vision loss.

We chose the ChatGPT-3.5 version because it is the free version and the most widely used version. The more advanced Chat-GPT-4.0 is a paid version released on March 13, 2023. Apple's Siri was not used because it only provided websites.

The four questions taken from the AAO section on “Floaters”^17^ are: (1) What are floaters? (2) What are flashes? (3) Flashes and Migraines? (4) Floaters and Flashes Treatment?

## Data analysis

For scoring the reading level, we used the “Flesch–Kincaid Grade Level Formula” which presents a score as a U.S. grade level.^21^ This test is used in the U.S. Department of Education to allow teachers, parents, librarians, and others to judge the readability level of school books and textbooks. The formula yields the “mean number of years of education” generally required to understand the book, text, or assigned reading. The resultant score (grade level) is particularly relevant when the number is greater than 10th grade. The formula for Flesch–Kincaid is^21^:

Grade Level = 0.39 × (words/sentences)  +  11.8 × (syllables/words) − 15.59

The AAO.org website videos were transcribed into Google Docs, using the “closed caption” option. The transcribed English language responses were pasted into WebFx.com^21^ to obtain the reading levels.

For the word count measurement, we employed Google Docs tools. The transcription of the video was retyped into Google Docs for word count.

The 22 keywords from the AAO website were used to score ChatGPT-3.5, Bard, Google Assistant, and Alexa's responses.

## Statistical analysis

The Mann-Whitney U test was used for all analyses. The scores of the Flesch–Kincaid tests were analyzed by Mann Whitney U test (Table 1). The word count was evaluated for significance (Table 2): (1) AAO vs LLMs, (2) AAO vs virtual assistants, (3) AAO vs LLMs + virtual assistants, and (4) LLM vs VA.

**Table 1.** Flesch–Kincaid grade level for Bard, ChatGPT, Google Assistant, Alexa, and AAO.

Question No.	Flesch–Kincaid Grade Level				
	ChatGPT	Bard	Google	Alexa	AAO
1	12	7.5	18.2^§^	7.8	7.5
2	9.1	8.7	8.8^†^	9.7	7.6
3	14.4^§^	12.4	N/a^‡^	6.7	4
4	13.7^§^	10.2	12.3	8	10.1
**Average**	12.3	9.7	13.1^§^	8.1	7.3
**Standard Dev**	2.4	2.1	4.8	1.2	2.5

LLM: large language model; AAO: American Academy of Ophthalmology.

† Question 2 for Google Assistant has a nonmedical definition of “flashes.”

‡ Question 3 for Google Assistant has a blank response.

§ College level.

AAO vs LLM: *p* = .11; AAO vs VA: *p* = .34; LLM vs VA: *p* = .69; AAO vs LLM + VA: *p* = .11.

**Table 2.** Word count for ChatGPT, AAO.org, and Google Assistant.

Question No.	Word count				
	ChatGPT	Bard	Google	Alexa	AAO
1	166	235	25	139	186
2	216	220	86^†^	34	71
3	197	306	N/a^‡^	25	85
4	242	229	45	55	383
**Average**	205.3	247.5	52.0	63.3	181.3
**Standard Dev**	32.0	39.5	31.1	52.0	143.9

LLM: large language model; AAO: American Academy of Ophthalmology.

† Question 2 for Google Assistant has a non-medical definition of “flashes.”

‡ Question 3 for Google Assistant has a blank response.

AAO vs LLM: *p* = .34; AAO vs VA: *p* = .057; LLM vs VA: *p* = .0286, AAO vs LLM + VA: *p* = 1.0.

## Results

Google Assistant uses “ophthalmologist” once whereas ChatGPT, Bard, and Alexa make no mention. The AAO website mentions the word three times. There are 22 AAO keywords. Keyword inclusions are ChatGPT 8, Bard 9, Google Assistant 4, and Alexa 3, with an average of six keywords (27.3%) vs AAO 22/22 (100%). Weighting the keywords in the four questions, ChatGPT, Bard, and Google Assistant, Alexa scores are 29/100 (29%) vs 100% of the AAO score (Table 3).

**Table 3.** Comparison of keywords and weighted score for Bard, ChatGPT, Google Assistant, Alexa, and AAO.

Qn	Comparison of keywords											Weighted score					
	AAO terms	Bard	CGPT	Google	Alexa	AAO	Bard total	CGPT total	Google total	Alexa total	AAO total	Points	Bard points	CGPT points	Google points	Alexa points	AAO points
**1**	**Vitreous**	✓	✓	✓	✓	✓	**2**/**6**	**2**/**6**	**2**/**6**	**1**/**6**	**6**/**6**	**6**	**12**/**25**	**12**/**25**	**12**/**25**	**6**/**25**	**25**/**25**
	**Retina**	✓	✓	✓		✓						**6**					
	**Posterior vitreous detachment**					✓						**10**					
	**Nearsighted**					✓						**1**					
	**Had cataract surgery**					✓						**1**					
	**Inflammation inside the eye**					✓						**1**					
**2**	**Stars**					✓	**3**/**4**	**¾**	**0**/**4**	**0**/**4**	**4**/**4**	**5**	**20**/**25**	**20**/**25**	**0**/**25**	**0**/**25**	**25**/**25**
	**Flashing lights**	✓	(Flickering)*			✓						**5**					
	**Lightning streaks**	✓	✓			✓						**5**					
	**Vitreous rubs/pulls on retina**	✓	✓			✓						**10**					
**3**	**Migraine**	✓	✓		✓	✓	**1**/**4**	**¼**	**0**/**4**	**1**/**4**	**4**/**4**	**5**	**5**/**25**	**5**/**25**	**0**/**25**	**5**/**25**	**25**/**25**
	**Migraine headache**					✓						**5**					
	**Ophthalmic migraine**					✓						**7**					
	**Migraine without headache**					✓						**8**					
**4**	**Floaters**	✓	✓	✓	✓	✓	**3**/**8**	**2**/**8**	**2**/**8**	**1**/**8**	**8**/**8**	**1**	**5**/**25**	**2**/**25**	**11**/**25**	**1**/**25**	**25**/**25**
	**Flashes**	✓	✓			✓						**1**					
	**Ophthalmologist**			✓		✓						**10**					
	**New floaters**					✓						**2**					
	**A lot of floaters**					✓						**2**					
	**Shadow appears in peripheral (side) vision**					✓						**3**					
	**Gray curtain covers part of vision**					✓						**3**					
	**Detached retina**	✓				✓						**3**					
**Total keyword appearance**							**9/22**	**8/22**	**4/22**	**3/22**	**22/22**	**Points earned**	**42**/**100**	**39**/**100**	**23**/**100**	**12**/**100**	**100**/**100**
**Ophthalmologist mentioned in number of times**							**0**	**0**	**1**	**0**	**3**						

*Flickering (included as a key term for ChatGPT).

**Figure 2.**
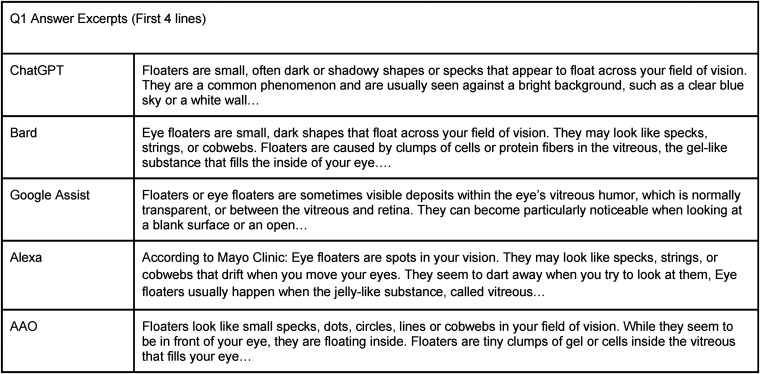
Excerpts of question 1 answers by Bard, ChatGPT, Google Assistant, Alexa, and AAO.

AAO.org has a reading comprehension of a lower grade level (average = 7.3 grade ± 2.5) that is different from the LLMs (*p* = .11). The reading comprehension value for LLM vs VA is not significant. Both require a higher reading level than the AAO website (*p*-value of VA + LLM vs AAO-use nonparametric Mann Whitney) (Figure 2).

AAO: American Academy of Ophthalmology.

In both ChatGPT and Google Assistant, there may be errors of omission as none mention “retinal specialist” since floaters can lead to a retinal detachment. Floaters and retinal detachments are usually treated by retinal specialists or ophthalmologists. ChatGPT's answers are conversational but contain redundancies without furthering medical knowledge. Google Assistant's answer to question two, “what are flashes” led to a nonmedical explanation of a flash (electricity) with no relevance to health or eye disease. For question 3, Google Assistant had no response to flashes and migraines, only a blank screen (Table 2). There were no “hallucinations” or answers that were nonsensical in our study.

## Discussion

In our study, we examined chatbots and virtual assistants’ answers to floater questions.^22,23^

Many people over age 40 have floaters due to aging, body trauma, and concussive injuries to the head. Risk factors such as myopia, diabetes mellitus, Lasik, and previous eye/retinal surgery play a role in the formation of floaters.

General practitioners are aware of the visual consequences when both flashes and floaters are present.^23,24^ The need to see an ophthalmologist is important because floaters can lead to retinal diseases such as diabetes and retinal detachment.^20,23,24^ In contrast, the LLM and virtual assistants do not emphasize the need to see an eye MD. In ChatGPT, words are strung together grammatically but there is no sense of causality, associated risk factors, or urgency of diagnosis.^25–28^ Thus, the patient could read the answer and feel that there is no need to see an ophthalmologist when there might be a retinal hole, diabetic vitreous hemorrhage, or vascular occlusion.

The chatbots are not programed for causality or inferential “thinking” but only for linking sentence fragments together.^25–28^

Given the word “floaters,” AI-mediated chatbots may predict the next word to be “vitreous” or “flashes of light” based on statistical parameters learned from prior training data sets on how often those words appear together.^25–30^ ChatGPT may need exposure to multiple and repetitive medical sentence fragments such as, “see a retinal specialist” associated with floaters in order for it to generate these interrelated concepts in its answers.^25–28^ Google's virtual assistant uses its 2016 version of AI technology and search engine optimization to find websites. To that end, the answers may lead to the National Eye Institute or the Mayo Clinic websites which may provide better answers to the patient with floaters than ChatGPT.

As ophthalmologists, we should be aware of the power of our words in our articles and websites as they will be used inadvertently in the “training” of ChatGPT, other chatbots,^1^ and virtual assistants.^25–30^ The frequent, hidden coding of the phrases, “see an ophthalmologist/retinal specialist” along with “floaters” may be a needed addition to our ophthalmic/retina websites and web journals.^25–30^

For clinical guidelines, AAO.org is excellent for patients.^17^ Its webpage on “Floaters and Flashes” ends with asking the patient to see an ophthalmologist if they are experiencing both floaters and flashes and details the risks of a retinal detachment. Ophthalmologists, General Practice Physicians, and the NIH website state the same in their literature.^24,31^ The AAO has guidelines and we as physicians should work with our colleagues in ehealth and software engineers to create an improved chatbot and AI-mediated virtual assistants for public education.^17^

In terms of ease of use, the virtual assistants ChatGPT and Bard are very easy to use. They require no prior knowledge when asking a question. They require an average of 2 to 3.18 s to access the prompt and begin typing. (GW, AW author testing). To find the website button for patients’ inquiries for the AAO website, it takes 13.05 s; similarly, to find the National Institutes of Health website, prompt for patients, it takes 7.69 s. For patients, LLMs are free and widely accessible. Similarly, Google Assistant has more robust answers than Amazon's Alexa. For general practitioners and ophthalmologists, the LLM models are not perfect but they do provide factual information. They do not direct the user to see an ophthalmologist in an urgent manner. If a person has new floaters with flashing lights, it is important for the person to see an ophthalmologist as quickly as possible to evaluate for retinal detachment. All physicians, generalists and specialists know the importance of early diagnosis of retinal detachment versus the ChatGPT response. The Bard response is now usually preceded by a caveat that it is not a physician.

## Limitations

There are limitations to our study on LLM chatbots and virtual assistants, due to the limited number of questions. In addition, the LLM chatbots are quickly evolving and changing. By the time this manuscript is printed, there will be new training by the chatbots and its algorithms and improvements will be made.

These AI models are trained on a large scale using existing data and if the data sets include biased information, the AI-generated answers will likely incorporate those biases unless a human intervenes. The issue of perpetuating biases raises public health and public policy issues about who should monitor the output and what values should AI models incorporate in their algorithms.

While AI models produce useful outputs, they will need humans, such as physicians or healthcare providers to optimize those “answers.” Originally, the AI models were not intended to reveal truth or display correct knowledge, instead, they were used to generate content or display words that were most likely to come next. Thus initially, the AI models “wrote poetry” or “wrote essays.” We cannot expect the AI-generated outcome to be necessarily true statements. These AI tools are known to “make things up” (hallucinate) and therefore cannot be expected to be used without audits for correctness. Users may have additional knowledge or context that the AI model does not, e.g., surgeons know the anatomy and have performed complex hernias and that context is not necessarily part of the training of the AI models.

## Conclusion

In conclusion, ChatGPT has language models available and accessible for clinical decision-making and patient education.^22,23^ As physicians, we need to be aware of ChatGPT, Bard, Google Assistant, and Alexa's capabilities and limitations as they may communicate possible inaccuracies and biases with their generative algorithms.^25–28^

Bard and PALM2 are both Google-directed AI LLMs.^22,28–30,32^ Bard was launched briefly in March 2023 and May 2023 with mixed results in Europe. However, in mid-August 2023, Bard was launched in the United States. Its algorithms are augmented with those created under PaLM2.^30^ As of this writing, the speed at which ChatGPT is gaining users has slowed due to the release of Bard which has 40 available languages.

PaLM2 has capabilities of 160 languages and future capability of “deductive reasoning.” It is in the “training” stage with Google engineers.^30^

We, as clinicians, may use them for patient education with caveats to our patients.^25–30,32^ It is crucial for physicians to recognize the influence of their written “phrases,” and “word associations” as these are used in the training of chatbots by their software programmers. Frequent inclusion of the phrase “see an ophthalmologist” may be needed in the software codes of ophthalmic websites and web journals, to ensure the inclusion of “ophthalmologists” in the AI vocabulary of AI-mediated chatbots.

ChatGPT's paid version (4.0) is earning robust revenues but the free version is still being used by the lay public and our patients. However, it is unclear as ChatGPT battles with Bard for supremacy in the space of Large Language Models, which one will have more downloads. Our patients have access to mobile apps, the internet, ChatGPT, Bard, Google Assist, and Alexa. Free chatbots and virtual assistants may be the first-choice solution when they seek free patient education. Google Assistant holds the largest market share of all virtual assistants for queries.

The use of these free chatbots can save the Retina Clinic time and money as labor resources become scarce with increasing patient load and electronic chart documentation demands. A medical assistant is paid an average salary of $37,190 in the United States in 2021, according to the Bureau of Labor Statistics.^33^ The cost of ChatGPT 3.5 is zero for the year. The ChatGPT can provide patient education with any mobile device. Its portability lends to ease of use when confronting a barrage of repetitive questions. One can imagine the cost savings of one fewer medical assistant with full-time benefits versus a free Chabot. At most, the paid version of ChatGPT 4.0 is $20 per month. The potential cost savings of these chatbots and virtual assistants may be an incentive for adoption for Retina offices. Capturing 100 million users in six weeks led Open AI, the creator of ChatGPT, to gain market share and crush its competitors. Open AI, by having a huge user base, will discourage others from trying to compete thereby creating a monopolistic system or at most, a duopolistic system, e.g., ChatGPT vs Google's Bard.

As physicians, we can assign medical assistants to show ChatGPT to selected patients with “floaters.” In some cases, patients want reassurance and repeated explanations of “what is a floater.” Then, these chatbots and virtual assistants are a cost-effective means of public education, with the proviso that there is a medical assistant to help them with the “factual blunders” or omission of the word, “ophthalmologist.” For informed consent, ChatGPT can translate and “write” in 95 languages. The retinal surgeon could use ChatGPT for translation services from the web or internet. ChatGPT's translations may be downloaded or made available by screenshots.

In the near future, ophthalmology and retina training programs may use chatbots to aid future trainees in the development of clinical reasoning and skills through generative case reports, similar to oral board examination questions about retinal floaters. ChatGPT, Bard, Google Assistant, and Alexa, when used together, may help our patients with disadvantaged socioeconomic backgrounds or linguistic, health literacy, and mobility problems.

To our knowledge, this is the first study to evaluate a common retinal condition with vision-threatening implications using chatbots and virtual assistants to answer questions from the national ophthalmology physician organization.^34–38^ As physicians and ophthalmologists, we can use the latest AI-mediated tools to further our mission: do no harm and improve patient education.
